# Cardiac Manifestations in COVID-19 Patients: A Focus on the Pediatric Population

**DOI:** 10.1155/2021/5518979

**Published:** 2021-07-16

**Authors:** Tania Abi Nassif, Ghina Fakhri, Nour K. Younis, Rana Zareef, Farah Al Amin, Fadi Bitar, Mariam Arabi

**Affiliations:** ^1^Department of Pediatrics and Adolescent Medicine, American University of Beirut Medical Center, Beirut, Lebanon; ^2^Faculty of Medicine, American University of Beirut Medical Center, Beirut, Lebanon; ^3^Division of Cardiology, Department of Pediatrics and Adolescent Medicine, American University of Beirut Medical Center, Beirut, Lebanon

## Abstract

**Background:**

SARS-CoV-2 is a new strain of the coronavirus family that emerged by the end of 2019 and led to the unpreceded COVID-19 pandemic. The virus affects multiple organs simultaneously and leads to a high rate of morbidity and mortality in all age groups. The cardiovascular system is one of the major affected organ systems. Various mechanisms including direct myocardial injury contribute to the cardiac manifestations of COVID-19 patients.

**Methods:**

We performed a comprehensive and updated search on the cardiac manifestations of COVID-19. Our search included laboratory and imaging evaluations. In addition, we added a unique section on the effect of SARS-CoV-2 on the cardiovascular system in the pediatric population.

**Results:**

COVID-19 might have an effect on the cardiovascular system at various levels leading to myocardial ischemia, arrhythmia, heart failure, myocarditis, and multisystem inflammatory syndrome in children. The incidence of cardiovascular complications varies among patients. This paper also provides a comprehensive summary of all the reported pediatric cases with cardiac manifestations.

**Conclusion:**

Multidisciplinary teams are crucial for adequate management of patients with COVID-19 regardless of age. Timely diagnosis is critical in reducing mortality.

## 1. Introduction

Coronaviruses have previously recorded two large epidemics in 2003 and 2012, respectively [1, 2]. In December 2019, an outbreak of atypical pneumonia was witnessed in Wuhan, China. The causative agent of this outbreak was a novel coronavirus, named severe acute respiratory syndrome coronavirus 2 (SARS-CoV-2). Later, the World Health Organization (WHO) acknowledged the disease related to this virus as coronavirus disease-19 (COVID-19). In March of 2020, COVID-19 was declared to be a world pandemic [3].

SARS-CoV-2 is an enveloped positive-sense single-stranded RNA virus. It spawns a wide spectrum of diseases affecting multiple organs. By early May 2020, the virus had already reached over 200 countries worldwide afflicting at least 4 million victims. As of February 10, 2021, there have been more than 106 million confirmed cases of COVID-19 and more than 2 million deaths around the globe [3, 4].

Despite being of zoonotic origin, SARS-CoV-2 exhibits human-to-human transmission through the droplet, contact, and ocular routes. It can remain viable for 3 hours as an aerosol and for 72 hours on certain surfaces. The virus mainly invades the upper respiratory tract leading to respiratory symptoms. However, multisystem involvement is observed as well, including the cardiovascular system. Indeed, the natural history of COVID-19 proved that the cardiovascular system may act as a primary target for SARS-CoV-2 and thus may be influenced directly by the virus. Similarly, secondary sequelae of the disease provoke an indirect injury to the cardiovascular system. Therefore, it is reasonably valid that patients with underlying cardiovascular morbidities are at increased risk of experiencing severe disease and a higher mortality rate [5, 6].

The virus can directly invade cells of various origins including the pulmonary, cardiac, and intestinal cells. Subsequently, tissues are injured through direct viral invasion and indirect release of cytokines and inflammatory markers. The exaggerated inflammatory response accompanied by the cytokine storm is a key player in the multiorgan involvement of the disease. Initially, most patients present with fever, dry cough, and shortness of breath. As the disease progresses, patients may develop nausea, vomiting, abdominal pain, and diarrhea [6]. Around 10% of patients experience severe disease and require intensive care and supplemental oxygen therapy. These patients are at increased risk of developing acute respiratory distress syndrome with diffuse alveolar damage, cardiac injury, viremia, superimposed bacterial infections, and multiorgan failure [7].

Owed to the rapid development of events, a considerable amount of data has become available in the literature to explain the pathophysiology and clinical features of COVID-19. However, only a few reports provide a comprehensive review of the cardiovascular manifestations. Besides, when it comes to COVID-19 in the pediatrics population, the literature is scarce and limited. This is largely because children exhibit milder disease forms. In this paper, we provide a comprehensive review of the cardiovascular manifestations of COVID-19, with a special focus on the pediatric population.

## 2. Renin-Angiotensin-Aldosterone System

The renin-angiotensin-aldosterone system (RAAS) is a complex system involved in regulating blood pressure and maintaining fluid and electrolyte balance. It can aggravate or precipitate atherosclerosis leading to myocardial hypertrophy and fibrosis [8]. Renin cleaves angiotensin to angiotensin I (AT1) which is cleaved by angiotensin-converting enzyme (ACE) to angiotensin II (AT2). Two receptors for AT2 with similar affinities exert a wide array of different cascading effects. ACE-2, a monologue of ACE, cleaves AT2 back to angiotensin 1–7. Angiotensin 1–7 exerts anti-inflammatory, antiproliferative, and vasodilating effects. The family of coronaviruses has a well-established mechanism of binding to ACE2 receptor, thus mediating its proteolytic modification and entry into the cell [9]. The outer spike S-proteins are found abundantly on the surface of the virus bind with high affinity to ACE2 receptor [10]. The infection is initiated when the virus is internalized into the cells via clathrin-coated pits. Its viral RNA is subsequently released into the cytoplasm. Zhou et al. reported that SARS-CoV-2 can only enter ACE2-expressing host cells to initiate the infection process [11]. These findings suggest that a shared receptor, ACE-2, plays a role in both determining the fate of AT2 and mediating the cytotoxicity of SARS-CoV-2. Hence, AT2 may be potentially influenced by SARS-CoV-2 internalization and intracellular replication, and in a similar manner, SARS-CoV-2 pathogenicity may be modulated by AT2 fate.

## 3. COVID-19 Pathophysiology

The pathophysiology of COVID-19 is reflected by three phases. The first phase is triggered by viral penetration into the respiratory epithelium and is followed by cellular proliferation. The initial immune response is marked by the activation of monocytes and macrophages and is portrayed by mild symptoms. The next phase begins with pulmonary vasodilatation and increased vascular permeability. Leukocyte migration then ensues leading to fluid extravasation and pulmonary edema. Consequently, alveolar damage, hypoxemia, cardiac damage, and stress are precipitated. The final phase is characterized by a massive cytokine storm secondary to the exaggerated inflammatory response [12, 13].

### 3.1. A Hyperinflammatory State

**Figure 1:**
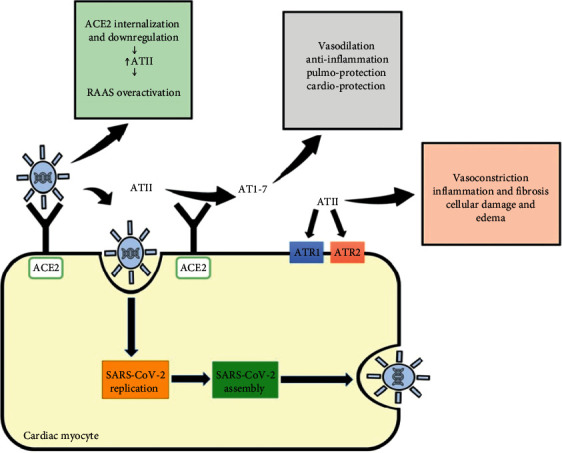
An Interplay between angiotensin II and SARS-CoV-2. SARS-CoV-2 cellular uptake is mediated primarily by the ACE2 receptor. Viral internalization is then followed by viral replication, assembly, and release. ACE2 is involved in converting angiotensin II (ATII) into angiotensin 1-7 (AT1-7). Unlike ATII, AT1-7 has anti-inflammatory, cardioprotective, and pulmoprotective effects with vasodilatory properties. Interestingly, SARS-CoV-2 binding to ACE2 provokes ACE2 internalization and downregulation. Consequently, this increases the circulatory levels of ATII and upregulates numerous proinflammatory, fibrotic, and vasoconstrictory pathways.

The excessive release of proinflammatory cytokines engenders a dramatically amplified immune response against the virus. Indeed, the plasma levels of interleukin-2 (IL2), interleukin-6 (IL-6), tumor necrosis factor *α* (TNF*α*), and C-reactive protein (CRP) are markedly elevated in COVID-19 patients. They are considered key contributors in the process leading to multiorgan failure [14]. Besides, the virus can downregulate the expression of ACE2 leading to a cascade of events and multiorgan manifestations summarized in Figure 1. The innate immune system plays a pivotal role in the uncontrolled inflammatory response mounted against the virus. The internalization and the reduction of expression of ACE-2 receptor on cellular surface result in enhanced levels of circulating Ang II. Ultimately, a vicious cycle, maintained by ever-increasing inflammatory markers, is developed [10, 15].

ACE2 is extensively expressed on the entire circulatory system, especially the coronary vessels. Vascular smooth muscles cells and endothelial cells present in both the arterial and venous systems express ACE2 on their surfaces. Viral entry and replication are potent triggers of an exaggerated immune response characterized by a cytokine storm and eventually by endothelial activation and dysfunction. The inflamed endothelium soon becomes dysfunctional and predisposes to a proinflammatory prothrombotic state [16].

## 4. SARS-CoV-2 and the Pediatric Population

The Chinese Center for Disease Control reported that <1% of the patients with COVID-19 were under the age of 10 years [12]. In another large pediatric-only cohort in China, authors reported that children tend to have a less severe form of COVID-19 with the majority presenting with mild symptoms. However, infants with COVID-19 were in a critical condition compared to older children [17]. The reason behind milder disease presentation in children is not yet clear. Some theories support the notion that children have a less functional ACE2 receptor compared to adults; thus, the infectivity of the virus is decreased. Another theory argues that children have higher levels of ACE2 which in its turn reduces Ang II plasma levels. Ang II is recognized to correlate strongly with the clinical course of COVID-19 disease [6]. Indeed, children infected with COVID-19 have low hospital admission rates and an estimated mortality rate of less than 5% [18]. However, a minority of children tend to exhibit serious disease manifestations, especially cardiovascular injury.  Table 1 depicts a comprehensive summary of the reported cardiac-related signs and symptoms of COVID-19 disease in children.

## 5. SARS-CoV-2 and the Heart

There are three mechanisms that lead to cardiac involvement in the setting of COVID-19: (1) direct injury caused by direct viral entry to myocardial cells, (2) hypoxia-induced myocardial ischemia, and (3) heightened exaggerated inflammatory response characterized by endothelial overactivation and microvascular thrombi [32, 33]. The virus exerts a direct effect on the myocardial cells by directly binding to the ACE2 receptors present on these cells leading to direct myocardial injury [34]. The beneficial role of ACE2 and angiotensin on the heart is well documented. Their interaction inhibits oxidative stress and cardiac remodeling and at the same time induces coronary vasorelaxation [35, 36]. It has been demonstrated that ACE2 expression increases at the initial phases of myocardial injury. In fact, ACE2 knocked-out animal models experienced exaggerated myocardial hypertrophy and heart failure. In COVID-19, there is a substantial downregulation of ACE2, and the subsequently elevated levels of angiotensin result in the overactivation of RAAS. Hence, the protective effect of angiotensin is lost, and myocardial injury is precipitated. Interestingly, elderly individuals are found to have lower levels of ACE2 expression, which could partially explain the poor outcome in this age group [37]. Moreover, there is a unanimous agreement that patients with underlying cardiovascular comorbidities are at a higher risk of contracting SARS-CoV-2 and developing severe disease. These patients also have significantly increased mortality rates [21, 38–41]. This is attributed to the reduced baseline cardiovascular reserve and to the ischemic injury prompted by the viral infection.

In a similar manner, the inflammatory markers correlate with electrocardiographic abnormalities and cardiac injury [42]. The elevation of inflammatory markers is accompanied by a surge in cardiac biomarkers and is associated with cardiac injury. Sequentially, worse clinical presentation and increased morbidity and mortality are encountered [43]. In a retrospective cohort in China earlier this year, the authors reported that the elevation of cardiac biomarkers was associated with increased mortality from COVID-19 [44]. Multiple studies have also demonstrated that patients with underlying cardiovascular diseases such as hypertension and coronary artery disease are at an increased risk to contract severe forms of COVID-19 and are at substantial risk of death. In a recent meta-analysis, Li et al. revealed that the relative risk of contracting severe COVID-19 and requiring intensive care was 2.03 (95% CI 1.54–2.68, *p* < 0.00001) and 3.3 (95% CI 2.03–5.36, *p* < 0.00001) in patients with hypertension and coronary artery disease, respectively [45].

Oudit et al. performed autopsies on patients with confirmed SARS infection. He reported that 35% of these patients had the virus's genome embedded in the DNA of the myocardial cells. The same subset of patients had a more aggressive course during their illness as compared to patients without cardiac involvement [46]. Edema of the cardiac stroma, atrophy of myocardial fibers, and inflammatory cells infiltration were also observed. Early acute involvement of the myocardial system was unanimously associated with higher morbidity and mortality [5–7, 45, 47]. Considering the strong homology between different types of SARS viruses, we can safely assume that SARS-CoV-2 may also invade the myocardial cells in a similar manner. Several studies have confirmed the presence of the SARS-CoV-2 genome integrated into the human myocardium [48–50]. In a recent report of 104 patients with COVID-19, endomyocardial biopsy confirmed that 5 of them had the SARS-CoV-2 genome in the myocardial tissues. Histologic examination revealed the presence of myocardial necrosis, inflammation, and microvascular thrombi [51]. In a study with a larger cohort, the authors documented the presence of the SARS-CoV-2 genome in almost 65% of the patients with COVID-19 [52]. Unanimously, it was found that a higher myocardial viral load is linked to a higher expression of proinflammatory cytokines [49–52].

### 5.1. Type I MI: Acute Myocardial Injury

Type I myocardial injury is defined by the acute elevation in cardiac troponin levels with or without evidence of myocardial ischemia on an electrocardiogram. The presence of acute myocardial injury among COVID-19 patients was identified as a significant factor associated with a 3.4-increased risk of death. Similarly, it is estimated that one-third to one-fifth of hospitalized patients with COVID-19 will have evidence of acute myocardial injury. Additionally, the presence of myocardial injury is also associated with a higher need for mechanical ventilation [53–55].

Myocardial infarction, under the umbrella of acute myocardial injury, is caused by the dislodgement of an atherosclerotic plaque in individuals with atherosclerosis. Viral products can predispose to plaque displacement by binding to and activating immune receptors on cells present within the plaque. This cascade of infection and inflammation causes coronary endothelial dysfunction and thrombosis [56, 57].

### 5.2. Type II Myocardial Injury

Type II MI emanates from an imbalance in myocardial oxygen demand and supply. In the setting of COVID-19, type II MI may arise from several contributory factors: (1) the presence of an atherosclerotic plaque that predisposes to gradual reduction in blood flow, (2) a dysfunctional endothelium, (3) elevated levels of ATII, (4) hypertension and vasoconstriction, and (5) hypoxemia [58–60]. During states of severe stress and increased inflammation, SARS-CoV-2 infection can lead to myocardial ischemia and infarction by disrupting the balance between myocardial oxygen supply and demand. This imbalance may be present with or without the effect of an atherosclerotic plaque, owing to the great physiological stress triggered by the infection.

The incidence of myocardial ischemia in the general COVID-19 population ranges between 7% and 40%, which reflects the heterogeneity of the studied population [14, 42, 43, 61].

Heart failure and myocardial damage contributed to approximately 40% of the deaths of SARS-CoV-2-positive patients. This mortality risk is significantly higher than the one associated with older age and prior medical diseases [44].

In adults, injury to the myocardium and subsequent troponin elevation was reported in up to 12% of SARS-CoV-2-infected patients [6, 62]. In another case series, the incidence of myocardial injury reached 7.2% [42]. In the pediatric population, myocardial injury in patients under 20 years of age was reported in terms of elevation of CK-MB, troponin, and Pro-BPN [19–21, 38–41, 63, 64]. In another large prospective study done exclusively on pediatric patients, at least 73% of the enrolled patients had elevated cardiac biomarkers. 50% of them had elevated troponin alone while the majority had increased Pro-BNP levels above 400 pg/mL [26].

In addition to the increased mortality risk, elevated troponin levels also correlate with a worse clinical picture (higher need for mechanical ventilation), as well as higher levels of other biomarkers such as IL-6, CRP, and D-dimer [32, 43]. Patients with myocardial injury and elevated troponin levels were consistently older than patients without myocardial injury. This phenomenon has not yet been reported in the pediatric population. The most common echocardiographic finding in patients with any element of myocardial injury is right ventricular dilatation and dysfunction. Left ventricular dysfunction is less commonly reported in the literature. Focal and diffuse ST-segment elevations are also common findings on electrocardiograms [65–67].

### 5.3. Heart Failure

Patients commonly present with tachycardia, which later leads to impaired diastolic filling and subsequent systolic and diastolic dysfunction. Eventually, mortality increases when patients present with hypotension and heart failure. A large cohort study reported that up to 7% of patients with SARS-CoV-2 had an element of left ventricular diastolic dysfunction with ensuing low cardiac output. On the other hand, in this same cohort, left ventricular systolic dysfunction was reported in more >50% of patients [63].

In one of the largest cohort studies in China limited to the pediatric population, 13 out of the 2135 patients who tested positive for SARS-CoV-2 had cardiac involvement [17]. Depressed ejection fraction is reported in most studies that assessed the cardiac function of the involved children. An ejection fraction as low as 10% was reported in one of the prospective studies in France [23]. In a large prospective study in pediatric patients with SARS-CoV-2, the authors reported that severe heart failure with ejection fraction <30% was present in 5% of the patients. Moreover, while the majority had an ejection fraction above 55%, ejection fraction declined to 30%–55% in a third of the children [26]. Another large prospective cohort study reported a degree of heart failure in the pediatric population. The mean of the left ventricular ejection fraction was found to be between 10 and 57%, with a median of 38% [23]. This suggests that SARS-CoV-2 may result in both diastolic and systolic myocardial failure not only in adults but also in pediatric patients.

### 5.4. Myocarditis

Myocarditis is defined as inflammation in the myocardium of the heart. Once present, it can cause dysfunction in the cardiac muscle, disruption in the electrical system, and reduction in cardiac contractility. The incidence is currently unknown in patients with COVID-19, as the evidence in the literature is limited to case reports [68–70]. In a cohort with small sample size, cardiac magnetic resonance imaging performed at a median of 71 days after the SARS-CoV-2 infection revealed myocarditis in 80% of the patients [71–73]. In the pediatric population, myocardial dysfunction has also been described. In a large prospective study in France, more than half of the children enrolled had evidence of myocarditis with depressed ejection fraction and elevated inflammatory and cardiac biomarkers. There was evidence of pericardial effusion as well [23]. Echocardiographic findings include a large left ventricular volume, left ventricular diastolic dysfunction, and low left ventricular ejection fraction. MRI findings often include a late gadolinium enhancement, a raised native T1 and T2, and pericardial enhancement [71].

In a large pediatric cohort study, myocarditis was noted in 71% of the admitted patients [23]. This suggests that SARS-CoV-2 induces myocarditis, myocardial ischemia, and heart failure in a significant proportion of the infected patients.

### 5.5. Arrhythmia

There are different types of arrhythmias described in the setting COVID-19 infection. The long list includes but not limited to sinus tachycardia, atrial arrhythmia, first-degree atrioventricular block, nonsustained ventricular tachycardia and fibrillation, premature atrial and ventricular beats, and incomplete right bundle branch blocks [21, 40, 41]. In the pediatric population, the following arrhythmias were reported: atrial arrhythmia, atrioventricular block, and bundle branch blocks [41]. The estimated incidence rate of arrhythmia in COVID-19 patients is around 17% [62]. The Heart Rhythm Society has recently pointed out that in addition to the direct myocardial injury and ensuing arrhythmia, electrolyte disturbances are quite common and can induce arrhythmias themselves. Despite these hypotheses, the exact mechanism behind the initiation of any kind of arrhythmia in the setting of COVID-19 infection is still unclear. In another large cohort, atrioventricular block occurred in 28.7% of adult patients hospitalized for COVID-19. Sinus tachycardia was reported in 19.6%. In general, arrhythmia was reported in more than 50% of the patients in one cohort [63]. On the other hand, in a larger case series, only 16.7% of the hospitalized patients developed arrhythmia [42]. In a more recent large prospective study, pediatric SARS-CoV-2 positive patients had an incidence rate of arrhythmias of 12% [26].

### 5.6. Cardiomyopathy

The severe systemic inflammation in COVID-19 can precipitate cardiotoxicity and lead to cardiovascular dysfunction. Patients with sepsis-induced cardiomyopathy tend to have elevated circulating levels of inflammatory markers such as IL-6 and TNF-*α* [74, 75]. In vitro studies have shown that exposure to elevated levels of IL-6 and TNF-*α* led to left ventricular systolic dysfunction and reduced contractility [76]. In another study, the incidence of cardiomyopathy was reported to reach 33% in patients infected with the virus [6]. In a large cohort, up to 10% of patients hospitalized for COVID-19 were reported to have tricuspid, aortic, or mitral regurgitation leading to hemodynamic changes [63]. Moreover, cardiac dysfunction was reported in 41.2% of patients hospitalized for COVID-19 in a large cohort study. While less commonly reported in the pediatric population, in several cohort studies, the authors reported the presence of left ventricular dysfunction in patients under the age of 20 years. Left ventricular dilatation, trace mitral regurgitation, and hypokinesia of the inferior left ventricular wall were particularly reported in these patients [38–40].

### 5.7. Multisystem Inflammatory Syndrome in Children (MIS-C)

In April 2020, the National Health Services in the United Kingdom reported the first case of MIS-C. At the time, it was a new combination of atypical Kawasaki disease and toxic shock syndrome under the umbrella of severe COVID-19. Later, the WHO and the United States Center for Disease Control and Prevention set criteria for diagnosis with overlapping features [77]. The pathophysiology involves a sequela of events. The first one is denoted by acute necrotizing arteritis characterized by neutrophils infiltrating the vessel walls. This is followed by the formation of an aneurysm within the coronary artery. Macrophages and T cell lymphocytes infiltrate the damaged vessel wall to begin a chronic form of vasculitis. Over the years, myofibroblast proliferation leads to coronary artery stenosis. In the early acute phase, myocardial edema can develop leading to myocarditis before evidence of an aneurysm. A transient left ventricular dysfunction may occur and lead to cardiovascular shock in some patients [73].

**Figure 2:**
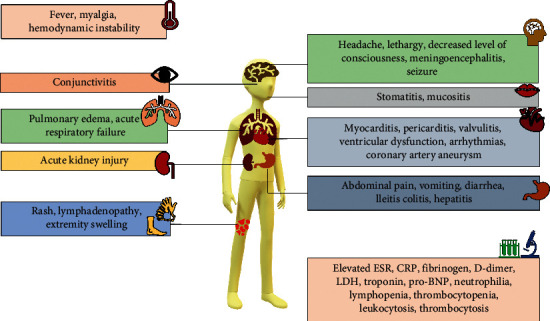
Clinical and laboratory manifestations of MIS-C. The most common and prominent features in patients with MIS-C include persistent fever, mucocutaneous manifestations, gastrointestinal symptoms, organ dysfunction, and significantly elevated inflammatory markers. The multiorgan effect of the SARS-CoV-2 is summarized in this figure.

Patients who develop MIS-C amidst their SARS-CoV-2 infection are usually older than patients who develop Kawasaki disease alone with the median age being 9 years in the former. The presentation tends to begin around 4–6 weeks after contracting the virus, and they are usually PCR-negative. They commonly report fever and a spectrum of respiratory symptoms ranging from cough to dyspnea. 70% of patients with MIS-C also report gastrointestinal symptoms such as abdominal pain and diarrhea. Other signs and symptoms that are related to the Kawasaki-like features are rash, fissured lips, and conjunctivitis [18]. The cardiac abnormalities common in MIS-C are arrhythmia, conduction abnormalities, ventricular dysfunction, coronary artery dilatation or aneurysm, and pericarditis. According to multiple reports, the most common cardiac abnormality is LV dysfunction manifested by a depressed ejection fraction.  Figure 2 contains a summary of the multiorgan involvement of SARS-CoV-2 in MIS-C.

While most patients are managed with inotropic support, a large cohort study revealed that around a third of the pediatric patients presenting with MIS-C required extracorporeal membrane oxygenation [64]. In a large case series, the majority of patients required inotropes but all of them recovered their LV function within a median of 2 days [72]. In another large pediatric-exclusive study including patients who developed MIS-C, the authors reported that 80% of them had cardiac involvement. The majority had elevated cardiac biomarkers (73%), and 8% developed coronary artery aneurysms [26].

Patients with MIS-C may develop coronary artery abnormalities such as dilatations and aneurysms. Reports have demonstrated an array of descriptions ranging from small aneurysms to giant ones. While it has been hypothesized that a mechanism similar to that observed in Kawasaki disease occurs with MIS-C, the true pathophysiology behind coronary artery abnormalities has not been elucidated yet [78].

In addition to coronary artery abnormalities, the second most common cardiac abnormality in MIS-C is arrhythmia. First-degree heart block is the most common presentation of arrhythmia. It has been commonly reported in children presenting with LV dysfunction. Electrocardiogram reveals QT prolongation, ST-segment changes, or T-wave abnormalities [79]. Laboratory workup reveals elevated troponin, Pro-Brain Natriuretic Peptide (Pro-BPN), D-dimer, and inflammatory markers such as ferritin, erythrocyte sedimentation rate (ESR), interleukin-6 (IL-6), procalcitonin, and C-reactive protein (CRP). A complete blood count reveals neutrophilia, lymphopenia, and thrombocytopenia [64]. Chest X-ray displays cardiomegaly with possible hilar and mediastinal abnormalities. More than 50% of these patients will have a degree of left ventricular dysfunction or failure noted on echocardiography. Global or wall hypokinesis has been documented as well. While right ventricular function is preserved in all patients, pericardial effusion is present in the minority of patients [22–31]. Only 15% of patients with MIS-C will have CAA noted at the time of presentation. T1- and T2-weighted cardiac magnetic resonance imaging reveals diffuse increased intensity consistent with myocardial edema [18]. The natural course of MIS-C is not yet known. Ventricular dysfunction tends to improve over time. More studies are needed to elucidate the long-term manifestations of MIS-C.

### 5.8. Cardiac Involvement in Heart Transplant Patients

Cardiac transplant patients are a vulnerable population whose immunosuppressed state poses a risk of severe forms of COVID-19 infection. Despite their scarcity, reported cases and series on transplant recipients with COVID-19 show a heterogeneous clinical spectrum ranging from mild presentations to more severe forms [80–82]. The therapeutic rationale lies in maintaining a chronic immunosuppressed state while dampening the viral infection. Decreasing the dose of immunosuppressant drugs upon diagnosis is still debatable [81]. In one single-center series by Caraffa et al., 5 adult cardiac transplant patients hospitalized with SARS-CoV-2 received a 50% reduction in their immunosuppressant drug dose in addition to a medium dose of corticosteroids and antibiotics. One possible hypothesis is that the complete suspension of immunosuppressive drugs accompanied by viral-induced hyperinflammatory response may reactivate the immune memory causing potential graft rejection [80]. In another case series of 4 heart transplant patients, symptoms were mild and like the general population despite their immunosuppressed conditions. No adjustments of immunomodulatory drugs were made in this age group [81]. In a cohort study of 47 heart transplant recipients affected by COVID-19 in Northern Italy, 80% of the patients required hospitalization with adjustment of their immunosuppressive medication dose. Both the infection prevalence and case-fatality rate (29%) were 2-fold higher compared to the general population. No myocarditis was observed in any of the patients [80]. Latif et al. reported a similar case-fatality rate (25%) in their cohort [83]. A survey in Wuhan, China, including 87 heart transplant patients reported a similar risk of SARS-CoV-2 infection as the general population provided preventive measures are properly practiced [84].

## 6. Conclusion

Cardiovascular involvement incurs significant prognostic value to patients diagnosed with COVID-19. Multidisciplinary teams with cardiology services are essential for managing all complications in patients suffering from COVID-19, as well as in patients with underlying cardiac diseases who are prone to severe and critical COVID-19. Ultimately, we argue that allocating sufficient resources for timely diagnosis and management of cardiac complications is crucial in reducing morbidity and mortality secondary to cardiovascular sequelae. In addition, performing a thorough evaluation with an electrocardiogram, echocardiography, and basic cardiac workup is essential for timely diagnosis and treatment.

## Figures and Tables

**Table 1:** Cardiac-related clinical descriptions of pediatric patients with COVID-19.

Reference	Number of cases	Country	Age	Sex	Physical exam	ECG	Echo	Elevated labs	Imaging	Treatment	Outcome
[19]	1	China	55 d	F	Tachycardia, respiratory distress, pharyngeal hyperemia, cough	NA	NA	Troponin, CK-MB, procalcitonin	Ground-glass opacities, pneumonia	IVIG, inhaled interferon alfa 1b, glutathione, Chinese lotus qingwen	Survived
[20]	1	China	13 m	M	Cough, crackles	NA	Heart failure	CRP, creatinine kinase, D-dimer, IL6, interferon-gamma,	Multiple patch-like shadows, pneumonia	Virazole, oseltamivir, interferon, IVIG, steroids, oxygen therapy, mechanical ventilation	Survived
[21]	9	China	11 m-10 y	3M	Productive cough	NA	NA	CK-MB, Pro-BNP, D-dimer	Pulmonary consolidation, ground-glass opacities	Inhaled interferon, ribavirin, lopinaviritonavir	NA
[17]	13/2135	China	0–18 y	12M	NA	NA	Heart failure	NA	NA	NA	NA
[22]	3	USA	6–13 y	2M	Tachycardia, tachypnea, hypotension, systolic murmur, dyspnea	Sinus tachycardia	Depressed function, MR, flow reversal in descending aorta, pericardial effusion	Procalcitonin, D-dimer, fibrinogen, troponin, CRP, LDH	NA	IL6 inhibitor, IVIG, hydroxychloroquine	Survived
[23]	21	France	3–17 y	9M	Cough	QT prolongation, ST-segment elevation, ventricular arrhythmia	Coronary artery dilatation, myocarditis, pleural effusion, depressed ejection fraction	CRP, procalcitonin, IL6, Pro-BNP, D-dimer	Local patchy shadows, ground-glass opacities, interstitial abnormalities	IVIG, aspirin, corticosteroids, inotropes, oxygen support, mechanical ventilation	Survived
[24]	1	USA	6 y	F	Syncope, respiratory distress, hypotension	Junctional rhythm	Depressed ejection function, MR	CRP, procalcitonin, Pro-BNP, D-dimer	Diffuse patchy infiltrates	Aspirin, IVIG, inotropic support, oxygen support, ECMO	Survived
[25]	99	USA	0–20 y	53M	Hypotension, cough, shortness of breath, wheezing	NA	Ventricular dysfunction, pericardial effusion, CAA	Troponin, Pro-BNP, D-dimer, CRP, fibrinogen, ferritin, ESR	Opacities, pleural effusion	Corticosteroids, IVIG, oxygen support, inotropic support, ECMO	2/99 died
[26]	186	USA	0–20 y	115M	NA	NA	Coronary artery dilatation, CAA, depressed EF, pericardial effusion	Pro-BNP, troponin, D-dimer, fibrinogen, INR	NA	IL6 inhibitors, IL1Ra inhibitor, IVIG, corticosteroids mechanical ventilation, ECMO, vasopressor support, oxygen support	4/186 died
[27]	15	USA	3–20 y	11M	Cough, dyspnea, chest pain, tachycardia, hypotension	Ventricular tachycardia, and ectopy, diffuse ST elevation	Depressed LV/biventricular function, coronary artery ectasia and dilatation, cardiogenic shock	Troponin, Pro-BNP, fibrinogen, CRP, D-dimer, procalcitonin, IL6, IL8, TNF alfa	Ground-glass opacities, pleural effusion	IVIG, steroids, anakinra, remdesivir, inotropic support, anticoagulation, mechanical ventilation, ECMO, intraaortic balloon pump, tocilizumab, COVID-19 convalescent plasma,	1/15 died
[28]	5	France and Switzerland	2–16 y	18M	Respiratory distress, chest pain	Ventricular arrhythmia, nonspecific ST elevation	Cardiogenic shock, LV dysfunction and hypokinesis, coronary artery dilatation, pericardial effusion	Troponin, creatinine kinase, Pro-BNP, D-dimer, CRP, procalcitonin, IL6	NA	Inotropic support, mechanical ventilation, ECMO, IVIG, steroids, anakinra	Survival
[29]	4	USA	3–20 y	3M	Tachycardia	Low voltage nonspecific *T*-wave changes, right axis deviation, ST-segment elevation, QTc prolongation	Myocarditis, MR, depressed LV and RV functions, pericardial effusion	CRP, ferritin, troponin, pro-BPN, D-dimer, ferritin, fibrinogen	NA	IVIG, anticoagulation, tocilizumab, convalescent plasma, mechanical ventilation, ECMO	1/4 died
[30]	1	USA	6 m	F	Tachycardia, tachypnea	NA	Normal	ESR, CRP	Opacities	Aspirin, IVIG	Survived
[31]	156	France	5–11 y	77M	NA	NA	Myocarditis	NA	NA	Mechanical ventilation, inotropic support	1/156 died

## Data Availability

Data are available upon request.

## Abbreviations

CAA: Coronary artery aneurysm
CRP: C-reactive protein
D: Days
ECMO: Extracorporeal membrane oxygenation
EF: Ejection fraction
ESR: Erythrocyte sedimentation rate
F: Females
IL1Ra: Interleukin-1 Receptor antagonist
IL-6: Interleukin-6
IL8: Interleukin-8
INR: International normalized ratio
IVIG: Intravenous immunoglobulin
LDH: Lactate dehydrogenase
LV: Left ventricle
M: Males
MR: Mitral regurgitation
NA: Not applicable
Pro-BNP: B-type natriuretic peptide
RV: Right ventricle
TNF alfa: Tumor necrosis factor alfa
USA: The United States of America.
